# Vaccination of pigs with the S48 strain of *Toxoplasma gondii* – safer meat for human consumption

**DOI:** 10.1186/s13567-015-0177-0

**Published:** 2015-05-01

**Authors:** Alison Burrells, Julio Benavides, German Cantón, João L Garcia, Paul M Bartley, Mintu Nath, Jackie Thomson, Francesca Chianini, Elisabeth A Innes, Frank Katzer

**Affiliations:** Moredun Research Institute, Pentlands Science Park, Bush Loan, Midlothian, EH26 0PZ, Scotland UK; Instituto de Ganadería de Montaña (CSIC-ULE), León, Spain; Instituto Nacional de Tecnología Agropecuaria (INATA), EEA Balcarce, Argentina; Departamento de Medicina Veterinária Preventiva, Universidade Estadual de Londrina, Londrina, Brazil; Biomathematics & Statistics Scotland, The King’s Buildings, Edinburgh, EH9 3JZ, Scotland UK

## Abstract

As clinical toxoplasmosis is not considered a problem in pigs, the main reason to implement a control strategy against *Toxoplasma gondii* (*T. gondii*) in this species is to reduce the establishment of *T. gondii* tissue cysts in pork, consequently reducing the risk of the parasite entering the human food chain. Consumption of *T. gondii* tissue cysts from raw or undercooked meat is one of the main sources of human infection, with infected pork being considered a high risk. This study incorporates a mouse bioassay with molecular detection of *T. gondii* DNA to study the effectiveness of vaccination (incomplete S48 strain) in its ability to reduce tissue cyst burden in pigs, following oocyst (M4 strain) challenge. Results from the mouse bioassay show that 100% of mice which had received porcine tissues from vaccinated and challenged pigs survived compared with 51.1% of mice which received tissues from non-vaccinated and challenged pigs. The presence (or absence) of *T. gondii* DNA from individual mouse brains also confirmed these results. This indicates a reduction in viable *T. gondii* tissue cysts within tissues from pigs which have been previously vaccinated with the S48 strain. In addition, the study demonstrated that the main predilection sites for the parasite were found to be brain and highly vascular muscles (such as tongue, diaphragm, heart and masseter) of pigs, while meat cuts used as human food such as chop, loin, left tricep and left semitendinosus, had a lower burden of *T. gondii* tissue cysts. These promising results highlight the potential of S48 strain tachyzoites for reducing the number of *T. gondii* tissues cysts in pork and thus improving food safety.

## Introduction

The protozoan parasite *Toxoplasma gondii* (*T. gondii*) has the ability to infect all warm blooded mammals, including humans and livestock. Livestock are known intermediate hosts of *T. gondii*. Food animals such as pigs, sheep, goats and cattle become infected with the parasite, either from consumption of oocysts shed in the environment by the definitive host (felids), or in the case of pigs, from consuming other infected intermediate hosts such as rodents [1]. Following *T. gondii* infection, cysts form in the tissues of the animal (tissue cysts), these cysts contain the bradyzoite stage of the parasite and can survive for the lifetime of the host. The ability of tissue cysts to establish within food producing animals varies; cattle rarely have detectable tissue cysts, even in animals which have been experimentally challenged [2,3], whilst in sheep, pigs and goats, tissue cysts are more commonly identified [4]. Tissue cysts have a preference to establish in specific tissues of the host, such as liver, heart, brain, tongue, diaphragm, and skeletal muscle [5,6]. Viable parasites and tissue cysts have also been isolated from cuts of meat and meat products destined for human consumption from naturally and experimentally infected animals [7-11]. Consumption of raw or undercooked meat from animals containing tissue cysts is a main source of *T. gondii* infection in humans, with infected pork considered to be the major source of infection [12,13].

Outdoor reared pigs are more likely to become infected with the parasite compared with those reared indoors [14,15], with the main source of infection thought to be from consumption of oocyst contaminated feed, water and/or soil [16]. In addition, outdoor housing systems also allow pigs to come into contact with rodents and other wildlife species. Due to their omnivorous nature pigs will consume rodents or rodent cadavers as well as other small mammals and birds, which may be infected with *T. gondii* and several studies have demonstrated how rodent control programs can reduce *T. gondii* seropositivity in pigs [1,17].

Although clinical toxoplasmosis in pigs is rare, and certainly not a common enough problem to warrant the commercial use of a vaccine against the parasite, the formation of tissue cysts in the muscles of infected animals can pose a significant risk for food safety and is thought to be one of the most important sources of *T. gondii* infection for humans, particularly when pork is eaten undercooked or raw [18,19].

A vaccine which can reduce or eliminate the formation of infective tissues cysts in pigs would be beneficial for pork products intended for human consumption, reducing the potential public health risk from *T. gondii* infection. Previous research into the reduction of the formation of tissue cysts in pigs has included work using live and killed vaccine approaches [20-25]. It is currently unknown whether the commercially available vaccine Toxovax® (comprised of the S48 strain of *T. gondii* and used to protect against ovine abortion caused by the parasite), offers any protection against tissue cyst formation in livestock species. This lack of knowledge regarding the ability of S48 to protect against tissue cyst formation was highlighted, as a key knowledge gap, in a recent document produced for the Food Standards Agency [26]. The document stated that one of the gaps in the current knowledge relevant to a UK risk assessment was: “*Vaccines based on live attenuated strains of tachyzoites are effective in reducing morbidity in sheep but it is not known whether vaccination has any effect on the formation of tissue cysts*”. Therefore, to address this knowledge gap and potentially improve food safety, this study focused on the effect of vaccination of pigs with the S48 strain of *T. gondii* in order to reduce tissue cyst formation. The S48 strain was originally isolated from a case of ovine abortion in New Zealand, which after approximately 3000 passages in mice lost its ability to develop into tissue cysts (bradyzoites) in mice and oocysts in cats [27,28].

The objective of this research was to evaluate the effectiveness of this live attenuated strain of *T. gondii* (S48) in its ability to reduce viable tissue cysts within porcine tissues. A reduction, or indeed elimination, of viable tissue cysts in pork would make this food source safer for human consumption. In addition, another objective was to ensure that the S48 strain used for vaccination did not persist in the pig tissues. Finally, the research aimed to provide an insight into the predilection sites for the parasite within specific porcine tissues, including tissues used within the human food chain.

## Materials and methods

### Pig vaccination and challenge

A total of 18 pigs, six week old Large White/Landrace cross bred pigs (*Sus scrofa*) of mixed gender, were divided into four groups (G) depending on experimental challenge; G1 (*n* = 5) non-vaccinated and oocyst challenged animals, G2 (*n* = 5) vaccinated and oocyst challenged animals, G3 (*n* = 5) vaccinated non-challenged animals, G4 (*n* = 3) non-vaccinated and non-challenged animals. The five animals in G2 and G3 were vaccinated subcutaneously (SC) with 1.2 × 10^5^ S48 tachyzoites 4 weeks prior to experimental challenge (day 0) (see Table 1). Four weeks following vaccination (day 28) animals in G1 and G2 were orally challenged with 10^3^ *T. gondii* oocysts of the M4 strain. During the experiment all animals were fed using a commercial pig feed and water was available *ad libitum*. All animal procedures complied with the Animals (Scientific Procedures) Act 1986 and were approved by the Moredun Research Institute ethics committee.

**Table 1:** **Animal groupings for vaccination and** ***T. gondii*** **challenge**

**Group**	**Vaccination and/or challenge**		**Number of animals**
	**Day 0**	**Day 28**	
**1**	n/a	1000 M4 oocysts	5
**2**	1.2 × 10^5^ S48 tachyzoites	1000 M4 oocysts	5
**3**	1.2 × 10^5^ S48 tachyzoites	n/a	5
**4**	n/a	n/a	3

### Sampling and measurements

Rectal temperatures of all pigs were monitored daily for 14 days post vaccination. At day 28 all animals were micro-chipped, iDENTICHIP® with Bio-Thermo (Animalcare Ltd., York, UK), and temperature monitored for 14 days post challenge. Blood sampling was carried out weekly from days 0 to 70 of the experiment. Blood was collected from the anterior vena cava using a 2.7 mL S-monovette serum tube with an S-monovette 20G × 1.5” safety needle (Sarstedt, Leicester, UK). Blood was left to clot overnight at 4 °C, tubes centrifuged for 10 min at 2000 *g* and serum transferred to a sterile 1.5 mL tube. Serum samples were stored at −20 °C until required. All 18 pigs were euthanised six weeks post challenge (day 70 of the experiment), by electrical stunning followed by severing of the jugular vein and exsanguination. Tissues (brain, chop, loin, left tricep, left semitedinosus, heart, masseter, tongue and diaphragm) were collected at *post mortem* for DNA extraction, pathology and mouse bioassay.

### Mouse bioassay

Mouse bioassay is considered the gold standard for determining the viability of *T. gondii* tissue cysts [29]. Ninety Porton mice (a minimally inbred stain), were used for the mouse bioassay. Food and water was supplied *ad libitum* and animals clinically monitored twice daily. Tissues from pigs in G1 (unvaccinated and oocyst challenged) and G2 (vaccinated and oocyst challenged pigs) were used for the mouse bioassay. Tissues were separated into three different 50 g groups, each group was based on the tissue type and divided into the following; “Brain” (50 g of brain), “Food” (a 50 g pooled sample which included 12.5 g each of chop, loin, left tricep and left semitendinosus), and “Other” (a 50 g pooled sample which included 12.5 g each of diaphragm, heart, tongue and masseter). These tissues were digested with acid/pepsin using a method previously described [30]. The tissue homogenate was centrifuged for 10 min at 1200 *g*, the supernatant was poured off gently and the final pellet was resuspended in 3 mL sterile saline (which contained 400 μg/mL penicillin and 400 units/mL streptomycin). Three mice were intraperitoneally injected with 400 μL of each inocula. An additional 400 μL of the inocula was stored at −20 °C for subsequent DNA extraction.

Tissues from pigs in G3 (vaccinated and non-challenged) and G4 (negative control group animals) were not included in the mouse bioassay.

Mice that showed either signs of infection, or which survived until the end of the six week bioassay, were euthanised by cervical dislocation. Blood samples were taken and brain tissue collected from each mouse. Half of each brain was stored separately in a sterile vial containing 1 mL PBS for DNA extraction, whist the remaining half was placed in 1 mL 10% buffered formalin for pathological examination.

### Detection of *T. gondii* DNA from mouse brains following bioassay

DNA extraction followed by a *T. gondii* specific nested ITS1 PCR (n-PCR) was completed for all mice used in the bioassay (*n* = 90). DNA extraction (adapted from [31]) initially required the homogenisation of each brain sample, which was achieved by passing the material through an increasing gradient of fine gauge needles (18G, 21G and 25G needles). 900 μL of Nuclei Lysis Solution (Promega, UK). Each 400 μL of mouse brain homogenate was incubated overnight at 55 °C, then once cooled, 300 μL of Protein Precipitation Solution (Promega) was added, mixed and incubated on ice for 5 min. The mixture was then centrifuged at 13 000 *g* for 5 min and the resulting supernatant transferred to a 2 mL tube containing 900 μL of isopropanol. Each tube was mixed by inversion and incubated at −20 °C overnight. The DNA was pelleted by centrifugation at 13 000 *g* for 5 min, supernatant removed and DNA pellet washed with 600 μL 70% ethanol. To avoid contamination, a DNA extraction control was included within each batch of extractions. The DNA pellet was centrifuged a second time and any residual ethanol removed and the pellet briefly air dried, with final re-suspension in 200 μL sterile H_2_O. To identify the presence of *T. gondii* DNA, a *T. gondii* specific ITS1 PCR was used, as previously described [32]. The ITS1 PCR was completed in triplicate for each mouse brain DNA sample. A positive control (*T. gondii* RH DNA) and multiple negative controls as well as the DNA extraction control were included within each PCR run.

### Detection of *T. gondii* DNA from mouse bioassay inocula

DNA was extracted from a 400 μL aliquot of the acid/pepsin porcine tissue homogenate (as previously described within this paper for DNA extraction from homogenised mouse brains). The DNA generated was tested for the presence of *T. gondii* by two different molecular methods; a qPCR targeting the *T. gondii* 529 bp repeat element [10], and the *T. gondii* ITS1 n-PCR [32] which is described throughout this study. By using both of these methodologies, the molecular detection of the parasite can be compared between the different techniques. In addition, this also allows the results from the molecular detection of the parasite, using DNA from the inocula, to be compared to detection of the parasite within individual mouse brains from the bioassay (mouse bioassay vs. molecular detection from bioassay inocula).

### Molecular detection of *T. gondii* DNA from pig tissues

DNA was extracted and tested for the presence of *T. gondii* from the following porcine tissues: brain, chop, loin, left tricep, left semitendinosus, diaphragm, heart, tongue and masseter. Aliquots of 1 g of each tissue for all pigs (*n* = 18) were tested individually. DNA was extracted using Precellys tubes containing ceramic beads (Peqlab, UK), followed by the *T. gondii* n-ITS1 specific PCR (used to detect *T. gondii* DNA), using the methodology previously described [31,32]. Each PCR was carried out in triplicate, with a positive control (*T. gondii* RH DNA), multiple negative controls and DNA extraction controls included within each PCR run.

### Porcine *T. gondii* IgG enzyme linked immunosorbent assay (ELISA)

The porcine *T. gondii* IgG ELISA was adapted from a similar methodology described for detection of *T. gondii* IgG in sheep [33]. Flat-bottom 96 well polystyrene microtitration plates (MICROLON, 96K, F-form, medium binding, Greiner Bio-one, Germany) were coated with 0.1 mL (2.5 μg/mL) of *T. gondii* tachyzoite antigens prepared as previously described [34], diluted in 0.1 M carbonate buffer (pH 9.6) and incubated overnight at 6 °C. The plates were washed 3 times with PBS-Tween 20 (0.07 M PBS/ 0.05% Tween 20 (PBS-T)) and non-specific immune sites blocked by incubation for 1 h at 37 °C with 125 μL of PBS-1% bovine serum albumin (BSA). Control and test sera were diluted 1:100 in PBS-T-1% BSA and added to the microtitre plates in duplicate, 0.1 mL in each well, and incubated for 1 h at 37 °C. Positive and negative control sera (pool of three animals) were included in each plate. After washing, peroxidase-labeled anti-pig IgG antibody (Sigma A5670, diluted 1:10 000 in PBS-T-1% BSA) was added 0.1 mL in each well and incubated for 1 h at 37 °C. After washing, the peroxidase activity was revealed by adding 0.1 mL of substrate solution (SureBlue TMB Microwell Peroxidase Substrate, KPL, Gaithersburg, MD, USA), and the reaction was stopped by adding 0.1 mL of 2 M H_2_SO_4_, and the optical density (OD) was read at 450 nm in an ELISA microplate reader (MRXII, thermo Labsystems). The average OD-value for the blank controls on a plate was subtracted from the OD-values of the sera on each plate. For control of plate-to-plate variation, the same positive and negative control sera were included on every plate and a corrected OD value was calculated for each sample as described previously [35]. A serum sample was considered to be positive when OD-value of the serum sample is greater than OD mean (from negative sera obtained from all plates, *n* = 64 – negative sera from the current experiment) plus 2 standard deviation (SD from negative serum from all plates).

### Quantification of histopathological lesions and imunohistochemistry labelling of porcine tissues

Methodology was carried out as described by [11]. Briefly, during histopathological examination, the numbers of glial foci and perivascular cuffs were counted for each tissue. IHC labelled slides were examined for labelled *T. gondii*-like structures (tachyzoites and tissue cysts). An animal was considered positive by IHC when positive labelling of tissue cysts or tachyzoites were found in at least one of its tissue sections.

### Murine *T. gondii* IgG ELISA

The ID Screen Toxoplasmosis Indirect Multi-species ELISA kit (ID.vet, Montpellier, France) was used to detect IgG against *T. gondii* from mouse serum. The supplied manufacturer’s instructions were followed and plates were read at 450 nm using the same ELISA reader as previously described. An ELISA was valid if the mean value of the positive control OD (OD_pc_) was greater than 0.035 (OD_pc_ > 0.035), and if the ratio of the mean OD values for the positive and negative controls (OD_pc_ and OD_nc_), were greater than 3.5 (OD_pc_/OD_nc_ > 3.5).

The interpretation of the result was classed as percent seropositivity (SP). A sample with an SP value of 50% or higher was positive, a negative result was an SP of 40% or less, and the result classed as doubtful if the SP was between 40% - 50%.

### Statistical analysis

To account for the increased variability with the mean, the weekly data on OD-value obtained from ELISA of serum samples of pigs post-vaccination were transformed using square root transformation. A repeated measures model was fitted to the transformed data incorporating treatment group, time (week post-vaccination as a categorical variable) and interaction between treatment group and time as fixed effects. The model considered a first-order autoregressive correlation structure between observations for each pig. The data on weekly rectal temperature of pigs were also analysed using a similar repeated measures model. Possible biologically interesting comparisons of differences in mean values between treatment groups were obtained using two-sided probabilities for each comparison. These probabilities were then adjusted using a False Discovery Rate (FDR) approach [36] to take into account the multiple comparisons of means so that the overall FDR was less than 5%. All *p*-values in this paper refer to FDR-adjusted probabilities unless they are specified to be global *p*-values.

For the mouse bioassay, all 45 mice, that received tissues (15 mice each fed with brain, food and other tissues) from the pigs of the G2 group (vaccinated and oocyst challenged), survived until the end of the experiment (day 42), and therefore, all data in this group were censored. A total of 22 mice, inoculated with tissues from the pigs of the G1 group (unvaccinated and oocyst challenged), died at different intervals. A Kaplan-Meir survival curve was plotted to present the mean survival proportions of mice between the two groups and the equality between two survival curves was tested using G-rho family of test [37]. Similarly, the equality of survival distributions between a pair of tissues within G1 group was also tested considering pig as a stratum variable. To account for multiple comparisons, these probabilities were then adjusted using a False Discovery Rate (FDR) approach as discussed earlier.

To test the agreement of each of the two molecular tests used for detection of parasite DNA from bioassay inocula (*T. gondii* 529 bp qPCR and conventional PCR incorporating the *T. gondii* specific ITS1 region) with gold standard test (the detection of *T. gondii* DNA directly from mouse brains following bioassay), the data on discordant cells were tested using an exact test of a null hypothesis about the equal probability of success (*p* = 0.5) in a Bernoulli distribution. Additionally, the data from the contingency table were used to estimate the sensitivity and specificity of both molecular tests and corresponding exact binomial confidence intervals.

All statistical analyses were carried out using R software version 3.0.1 with appropriate R packages (stats, nlme, multcomp, survival, ggplot) [38].

## Results

### Clinical observations in pigs

**Figure 1:**
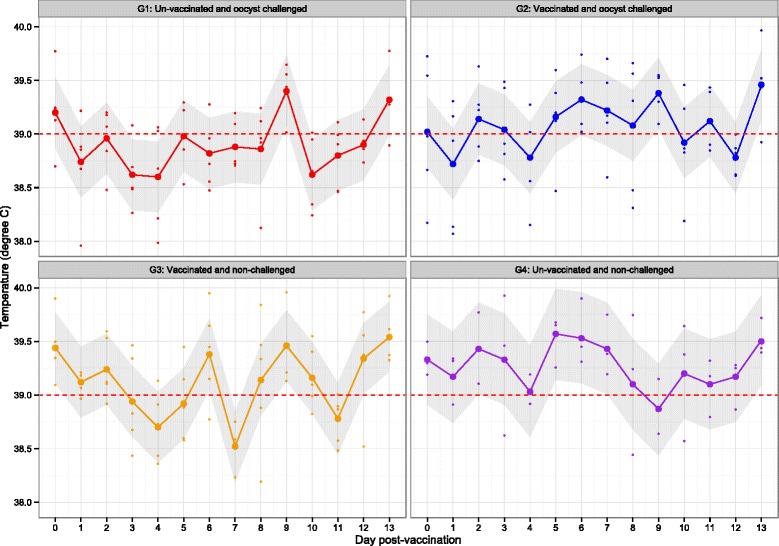
**Average porcine temperature per experimental group following vaccination with S48 tachyzoites.** Rectal temperatures of each pig was recorded daily prior to challenge. Between days 0 – 14 post vaccination, treatment groups G1 and G4, and G2 and G3 are identical. The red cut off line indicates the normal rectal temperature for pigs. G1 = Oocyst (M4) challenged pigs, G2 = Vaccinated (S48) and oocyst (M4) challenged pigs, G3 = Vaccinated (S48) pigs, G4 = Negative control pigs.

Rectal temperatures of all animals were recorded from days −5 to 14 of the experiment. Estimates of mean rectal temperature and 95% confidence intervals for four treatment groups along with observed temperature on each individual pig at each day post-vaccination (until day 14) are presented in Figure 1, although this can be noted in absence of oocyst challenge (which occurred on day 28), G1 & G4 and G2 & G3 are identical. Due to the increasing size of the animals, the technique used for recording the temperature from days 28 – 42 (daily for 14 days following oocyst challenge) was an implanted Thermochip®. However, this technique did provide accurate recording of the rectal temperature, probably due to the location of the chip. Temperatures fluctuated greatly resulting in vast variations of temperature readings. These readings were not reliable or informative, and therefore, were not used for further statistical analysis. On day 7 post-challenge (i.e. on day 35 of the experiment), all five animals in G2 appeared subdued/depressed for approximately 24 h, although they remained interested in food and movement was unaffected. All other animals remained clinically normal throughout the experiment.

### Molecular detection of *T. gondii* from individual pig tissues

DNA extracted from individual tissues (brain, chop, loin, left tricep, left semitendinosus, diaphragm, heart, tongue and masseter) from all pigs in G1, G2, G3 and G4 was tested for the presence of *T. gondii* DNA using the ITS1 n-PCR. Parasite DNA was not detected from any of these tissues, despite each DNA sample being tested in triplicate.

### Porcine *T. gondii* IgG ELISA

**Figure 2:**
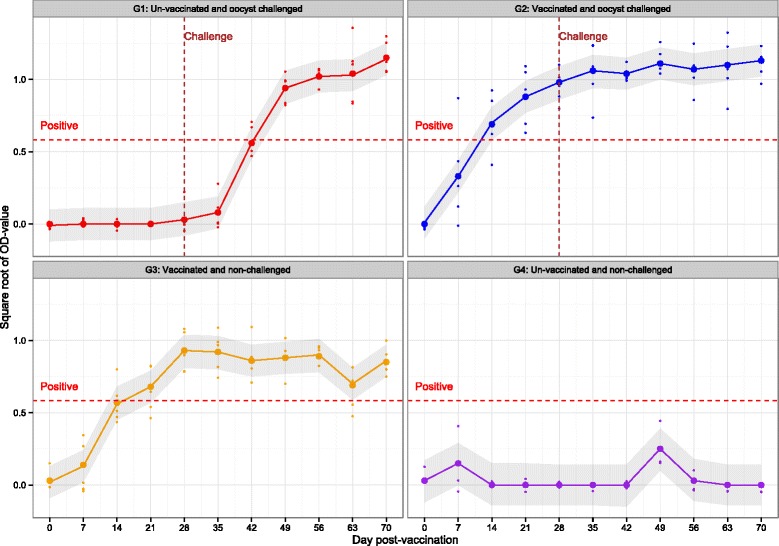
***T. gondii*** **IgG ELISA.** *T. gondii* IgG ELISA results for all pigs from week 0 to 70 post vaccination. Plots show observed ELISA value of each pig (small dots), estimated mean ELISA value of each group (large solid dots joined by solid line) and corresponding 95% confidence intervals (shaded region). The vertical dashed line indicates the point at which pigs were challenged with 1000 M4 *T. gondii* oocysts. The horizontal dashed line indicates when animals were classed as seropositive for *T. gondii*.G1 = Oocyst (M4) challenged pigs, G2 = Vaccinated (S48) and oocyst (M4) challenged pigs, G3 = Vaccinated (S48) pigs, G4 = Negative control pigs.

Estimates of the mean transformed OD values (square root transformation) and corresponding 95% confidence intervals for four treatment groups along with observed OD values on each individual pig at each week post-vaccination are presented in Figure 2. Briefly, all pigs tested negative by ELISA for *T. gondii* IgG at the beginning of the experiment (day 0 post-vaccination), however, by day 42 post-vaccination (or 2 weeks after challenge with *T. gondii* oocysts for G1 and G2), most of the pigs (apart from the pigs in G4 – negative control animals) were *T. gondii* IgG positive (OD-value greater or equal to 0.34, or equivalently, square root of OD-value greater or equal to 0.58; see Figure 2). The interaction effect of treatment group and time had a statistically significant (global *p* < 0.001) effect on the mean OD. The results showed that the mean OD value of pigs from G2 (pigs which were vaccinated with S48 and then challenged four weeks later with 10^3^ M4 *T. gondii* oocysts) were statistically significantly higher compared with G3 animals (pigs which were vaccinated and not challenged) at day 49 (*p* = 0.019), 63 (*p* < 0.001) and 70 (*p* = 0.017) post-vaccination (Figure 2). Mean OD values were higher in magnitude for the G2 group compared with G3 on day 42 and 70, but the mean differences were not statistically significant (*p* > 0.05). The negative control animals (G4) remained seronegative throughout the experiment as OD values were below the threshold during the entire experimental period (see Figure 2).

### Quantification of histopathological lesions and immunohistochemistry labelling of porcine tissues

Histological examination of the brain from the infected pigs showed mild non purulent perivascular infiltration, randomly distributed, in four animals from G1 and one animal from G2, mainly formed by CD3 positive lymphocytes. Besides these lesions, mild, focal, non-specific infiltration of few mononuclear inflammatory cells, mainly lymphocytes in the lungs and the different muscles studied were found in the all the animals studied. No differences were found between the studied groups regarding these non specific changes. Immunohistochemical labelling showed few intracellular *T. gondii* tachyzoites-like structures in the jejunal lymph node from one animal from G1 and in the retromandibular lymph node of another animal from G2.

### Mouse bioassay

**Figure 3:**
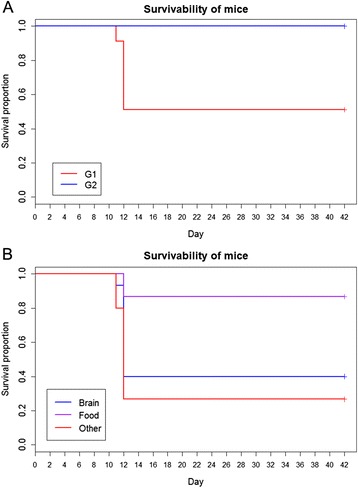
**Kaplan-Meir survival curves.** Kaplan-Meir survival curve in relation to vaccination highlighting proportion of mice that survived when fed tissues from G1 and G2 pigs **(A)**. All mice which received tissues from vaccinated and challenged pigs (G2) survived (blue line) until the end of the bioassay (day 42). Only 51.1% of mice which received tissues from unvaccinated and challenged pigs (G1) survived until the end of the bioassay (day 42). G1 = Mice inoculated with porcine tissues from pigs which were vaccinated (S48) and oocyst (M4) challenged. G2 = Mice which were inoculated with porcine tissues from pigs which were oocyst (M4) challenge. Kaplan-Meir survival curve from mice fed different tissues from pigs in G1 highlighting proportion of mice that survived when fed different tissue types (brain, food and other) **(B)**. Brain = brain tissue, Food = pooled sample of chop, loin, left tricep and left semitendinosus, Other = pooled sample of diaphragm, heart, tongue and masseter.

During the 6 week mouse bioassay 75.6% (68/90) of mice survived until the end of the experiment. Twenty two of 90 mice (24.4%) had to be euthanised due to the manifestations of clinical signs of *T. gondii* infection (ruffled coat, reluctance to move). All of these mice had received tissues from G1 pigs (unvaccinated and challenged with M4 *T. gondii* oocysts). From G1 mice, four animals were euthanised on day 11 of the experiment, and a further18 mice were euthanised on day 12, which left a total of 51.1% (23/45) of mice surviving until the end of the experiment (day 42) (see Figure 3A). In contrast, all mice, which received tissues from G2 pigs (vaccinated with S48 and challenged with M4 *T. gondii* oocysts), survived to the end of the experiment, resulting in 100% (45/45) survival of mice (Figure 3A). It was therefore not surprising that the independent two-sample log-rank test of the censored survival data until day 42 of the experiment showed strong evidence (*p* < 0.001), that mice which received porcine tissues from G2 pigs had a higher probability of survival across the entire range of the experimental period compared with mice which received G1 porcine tissues.

For mice that received tissues from G1 pigs, there was evidence that the mean proportion of mice that survived, differed between the porcine tissues (brain, food and other) that they were inoculated with (Figure 3B). Among them, 40% (6/15), 26.7% (4/15) and 86.7% (13/15) of mice survived when they were inoculated with “brain”, “other” and “food” tissues, respectively. Mice that received “food” tissues had a statistically significantly higher chance of surviving during the entire study period compared with mice that received “brain” (*p* = 0.018) and “other” (*p* < 0.001) tissues. The probability of survival between the mice that received “brain” and “other” tissues did not differ significantly (*p* = 0.340).

### Detection of *T. gondii* DNA from mouse brains following bioassay

**Figure 4:**
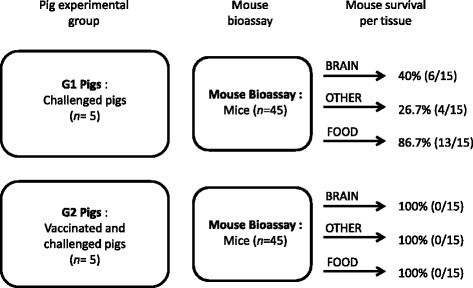
**Mouse survival rate related to specific porcine tissues (experimental groups G1 and G2).** All mice which received homogenised tissue from G2 pigs survived. Not all mice which received porcine tissues from G1 pigs survived, with only 40% of mice which received brain tissue surviving, followed by 26.7% which received food and 86.7% receiving other.

Brains from the 22 mice which were euthanised prior to the end of the six week mouse bioassay (all fed with G1 pig tissues – as shown in Figure 4), were positive by ITS1 n-PCR and *T. gondii* DNA was detected within the brains of all 22 mice. When DNA was extracted from the brains of the remaining 66 mice (which did not show any signs of *T. gondii* infection and were euthanised on day 42 of the bioassay), an additional two mice, which had been inoculated with brain tissue from G1 pigs, were identified as ITS1 positive. Therefore, *T. gondii* DNA was detected in 53.3% (24/45) of mouse brains which had been inoculated with tissues from G1 pigs (see Table 2). In contrast, 100% (45/45) of mice, which had been inoculated with tissues from G2 pigs, tested negative for *T. gondii* by ITS1 PCR (see Table 2).

**Table 2:** **Summary of** ***T. gondii*** **DNA detected by ITS1 PCR in mouse brains following bioassay with porcine tissues**

**Mouse Group**	**ITS1 positive mice (n)**	**% ITS1 positive**	**ITS1 negative mice (n)**	**% ITS1 negative**
**1**	24^a^	53.3	21	46.7
**2**	0	0	45	100
**TOTAL**	24	26.7	66	73.3

^a^ = including two mice which survived until the end of the end of the experiment (42 dpi).

1 = mice inoculated with porcine tissues from oocyst challenged pigs.

2 = mice inoculated with porcine tissues from vaccinated and oocyst challenged pigs.

**Table 3:** **Detection of** ***T. gondii*** **DNA from bioassay inocula (homogenised tissues) compared to detection of** ***T. gondii*** **DNA from mouse brain (bioassay) from vaccinated and oocyst challenged pigs (G2)**

**Pig No.**	**Brain**			**Other**			**Food**		
	**qPCR**	**ITS1**	**Bio**	**qPCR**	**ITS1**	**Bio**	**qPCR**	**ITS1**	**Bio**
**820**	−	−	−	−	−	−	−	−	−
**821**	−	−	−	−	−	−	−	−	−
**822**	−	−	−	−	−	−	−	−	−
**823**	−	−	−	−	−	−	−	−	−
**824**	−	−	−	−	−	−	−	−	−
**Positives**	0/5	0/5	0/5	0/5	0/5	0/5	0/5	0/5	0/5

Results from the 529 bp *T. gondii* qPCR (qPCR), conventional *T. gondii* ITS1 PCR (ITS1) and ITS1 PCR from mouse brain – bioassay result (Bio), from vaccinated and oocyst challenged pigs (G2).

### Detection of *T. gondii* DNA from bioassay inocula

Homogenised and acid/pepsin digested pig tissues used as inocula to challenge the mice were tested for the presence of *T. gondii* DNA. All inocula (*n* = 30) were tested (“food”, “brain” and “other” per pig, giving 15 inocula per experimental group), using both the *T. gondii* ITS1 PCR and the *T. gondii* 529 bp qPCR. *T. gondii* DNA could not be detected in any of the inocula (0/15) generated from G2 pigs by either of conventional *T. gondii* ITS1 n-PCR, or 529 bp qPCR (Table 3). The results from the ITS1 n-PCR showed that 7/15 (46.7%) of inocula comprised of tissues from pigs in G1 were positive for *T. gondii* DNA (Table 4). Of the seven positives, four were from the inocula containing “brain” tissue, two from the inocula comprised of the “other” tissues and one from the inocula comprised of the “food” tissues (Table 4). However, among inocula generated with tissues from G1 pigs, detection of *T. gondii* DNA using the qPCR was slightly lower compared with detection using the ITS1 n-PCR, with 6/15 (40.0%) of inocula testing positive by qPCR (compared to 7/15 (46.7%) by ITS1 PCR) (see Table 4). Inocula, which were used for bioassay in mice, with subsequent detection of *T. gondii* DNA directly from mouse brain, detects the greatest number of *T. gondii* positive tissue samples, resulting in 9/15 (60%) of pig tissue inocula testing positive. Four of these positives were detected in inocula comprised of “brain”, four from “other” and one from “food” tissues. Considering the data on bioassay results, there was however no evidence that mean proportions of detectable infected and non-infected samples differed between tissues (global *p* = 0.201).

**Table 4:** **Detection of** ***T. gondii*** **DNA from bioassay inocula (homogenised tissues) compared to detection of** ***T. gondii*** **DNA from mouse brain (bioassay) from oocyst challenged pigs (G1)**

**Pig No.**	**Brain**			**Other**			**Food**		
	**qPCR**	**ITS1**	**Bio**	**qPCR**	**ITS1**	**Bio**	**qPCR**	**ITS1**	**Bio**
**825**	+	+	+	−	−	−	−	−	−
**826**	+	+	+	+	−	+	−	−	−
**827**	+	+	+	−	−	+	−	−	−
**828**	+	+	+	−	+	+	−	−	−
**829**	−	−	−	−	+	+	+	+	+
**Positives**	4/5	4/5	4/5	1/5	2/5	4/5	1/5	1/5	1/5

Results from the 529 bp *T. gondii* qPCR (qPCR), conventional *T. gondii* ITS1 PCR (ITS1) and ITS1 PCR from mouse brain – bioassay result (Bio), from oocyst challenged pigs (G1).

### Comparison of molecular detection methods for detection of *T. gondii* DNA using bioassay inocula

Two molecular tests (*T. gondii* 529 bp qPCR and the *T. gondii* specific n-ITS1 PCR) were compared for their detection of parasite DNA from bioassay inocula using a gold standard test. The detection of *T. gondii* DNA directly from mouse brains following bioassay was considered as the “gold standard” as parasite DNA was detected in 9 out of 15 samples (60%) (see Table 4). The qPCR detected 6 out of 15 samples (40%), compared with 7 out of 15 samples (46.7%) by ITS1-PCR. Results showed that both qPCR (*p* = 0.250) and ITS1-PCR (*p* = 0.500) are in agreement with the gold standard test. However, the estimate of sensitivity of the ITS1 PCR (0.78 with 95% confidence interval: 0.40, 0.97) was higher compared with the 529pb qPCR (0.67 with 95% confidence interval: 0.30, 0.93). The estimates of specificity and 95% confidence interval for both tests were identical for both methods (1.00; 0.77, 1.00).

### Mouse serology and pathology

Serum samples from all mice (*n* = 90) were collected at *post mortem* and tested for the presence *T. gondii* IgG antibodies using the commercially available ID vet ELISA. The majority of mice (98.9%, 89/90) tested seronegative (with seropositivity (SP) ≤ 3%), which included all 22 mice euthanised due to signs of *T. gondii* infection. One mouse (C11-1), which was inoculated with brain tissue from a pig G1 pig, tested positive by ITS1 PCR and survived until the end of the experiment (day 42), was classified as seronegative (23% SP). However, the percentage seropositivity of this mouse was much higher than that of the remaining 89 seronegative mice. Only one mouse (C11-2), which was also inoculated with brain tissue from a pig in G1, tested positive for *T. gondii* IgG (189% SP, this mouse was euthanised at the end of the experiment (day 42) and tested positive by ITS1 PCR.

The pathological results completed on half of each mouse brain (*n* = 90) could not identify any differences between mice inoculated with different porcine tissues (brain, food and other), with the exception of three animals, where tissue cysts were observed (C11-2, C14-3 and C18-2), which had been inoculated with tissues from pigs in G1.

## Discussion

The results from this work clearly show that vaccination with a live attenuated strain of the parasite (S48) and subsequent challenge with *T. gondii* oocysts (M4) in pigs is successful in significantly reducing infective tissue cysts from establishing within porcine tissues, as shown by the mouse bioassay. Mouse survival rates from the bioassay at day 42 pi show a clear difference between those mice inoculated with tissues from pigs in G1 (unvaccinated and oocyst challenged pigs), compared with mice inoculated with tissues from pigs in G2 (vaccinated and oocyst challenged pigs) (Figure 3A). In our experience, infection with the M4 strain (type II) of *T. gondii* is likely to cause clinical signs in Porton mice, where viable tissue cysts present in the inocula (homogenised porcine tissue), generally results in severe clinical signs in mice and euthanasia is required. Therefore, in this study, reporting of mouse survival was important as it was a good indicator of the presence or absence of viable tissue cysts. It has previously been reported that as little as one tissue cyst (containing possibly thousands of infective bradyzoites) is enough to cause infection [39]. Other research, which has studied the vaccination of pigs to reduce tissue cyst burden, has mainly focused on microscopic identification of tissue cysts and/or detection of parasite DNA from mice used in the bioassay [21,23-25,35,40,41]. In these studies there is no detailed information about mouse survival and clinical signs of *T. gondii* during the bioassay itself. In fact, very few *T. gondii* studies, which incorporate mouse bioassays to assess the viability of tissue cysts, describe mouse survival rates in any detail. However, this might also be related to the strain of mouse used, as Swiss Webster mice (although susceptible to *T. gondii*) are generally thought to be more resistant to the parasite and are less likely to show clinical signs of infection. Research by Pena et al. [42], have reported mouse mortality rates following bioassay of cat tissues in relation to the genotype of *T. gondii* present. Our current research describes mouse survival rates using a Kaplan-Meir survival curve. Mouse survival provides an indication of the viability of *T. gondii* tissue cysts, however, this data is lacking in the majority of studies which incorporate mouse bioassay.

In addition to mouse survival rates, *T. gondii* DNA was not detected in any mice (0/45) following challenge with inocula comprised of porcine tissues from G2 pigs (vaccinated and oocysts challenged pigs) (Table 3), whilst parasite DNA was detected in 53.3% (24/45) of mice following challenge with inocula from G1 pigs (non-vaccinated oocyst challenged) (Table 4). Other research, investigating vaccination and *T. gondii* challenge of pigs and subsequent mouse bioassay, have focused only on the microscopic detection of tissues cysts from mouse brain following bioassay [18,21,25,41], rather than detection of *T. gondii* DNA from the mouse brain. Therefore, as the pathology results from the current research provide limited information, it is difficult to draw direct comparisons between these vaccination and challenge experiments and the current research. However, as 100% of mice from the bioassay survived following inoculation with tissues from pigs vaccinated and oocyst challenge, vaccination with S48 tachyzoites appears to be a very promising approach for reducing parasite burden in porcine tissues. Previous research into vaccination of pigs against *T. gondii*, which have incorporated mouse bioassay, have not shown such a protective response against tissue cyst formation in mouse brains. For example, following immunisation of pigs with crude *T. gondii* rhoptry proteins with Quil-A as an adjuvant, da Cunha et al. [41] found only partial protection from formation of tissue cysts in mouse brains following bioassay with porcine tissues, with the parasite detected in 5/11 (45.4%) mice in the vaccinated and challenged group. In a similar experiment, partial protection was observed by Garcia et al. [21], who used *T. gondii* rhoptry proteins and immunostimulating complexes (ISCOMS) as an adjuvant to vaccinate pigs. Dubey et al. [25] tested a vaccine incorporating a low dose of irradiated *T. gondii* oocysts, and although fewer tissue cysts were observed in mice, cysts were detected in 45/110 (40.9%) of mice which had received porcine tissues from vaccinated pigs. In other work by Dubey et al. [18] using tachyzoites of a non-persistent strain of *T. gondii* (RH) to vaccinate pigs, only partial protection was described, with fewer tissues cysts in mice inoculated with porcine tissues. Similar results were also reported using the *T. gondii* TS-4 mutant to vaccinate pigs [43,44], where the vaccine alone did not persist, however only partial protection was observed following challenge with GT-1 oocysts. In summary, apart for the research described within this manuscript, none of the literature currently available can describe 100% protection against tissue cyst formation in mice, following bioassay of porcine tissues.

From the current results, it appears that vaccination with S48 alone does not induce tissue cyst formation in porcine tissues, as results from the mouse bioassay, which included inocula with porcine tissues from the vaccinated and challenged animals, were all PCR negative for *T. gondii*. If a positive had been obtained from this group, the experiment had been designed to verify whether infection was due to vaccination (S48) or oocyst challenge (M4), as previously shown in lambs [11] (both S48 and M4 are different *T. gondii* genotypes; S48 = type I, M4 = type II). However, a bioassay using porcine tissues from animals that were vaccinated alone (which was not included in this study), could further support this result.

The results also show that there were differences in survival rates within groups of mice which were inoculated using tissues from G1 pigs, depending on which type of tissue they had been inoculated with (Figure 4). A greater proportion of mice survived which had received porcine inocula from the “food” tissue group (chop, loin, left triceps and left semitendinosus), compared with mice which had received tissues from the “brain” and “other” tissue groups (diaphragm, heart, tongue and masseter). This suggests that there are a greater number of viable parasites within tissues from the “brain” and “other” groups in comparison to tissues that are used routinely for human consumption (“food”). To further confirm this result, *T. gondii* DNA was detected in the brains of all mice which were euthanised due to clinical signs of *T. gondii* infection, with parasite DNA being detected in fewer bioassay mice which had received porcine tissues from the “food” group using tissues from G1 pigs (oocysts challenged pigs), compared with mice which received brain or tissues from the “other” group (Table 4).

In addition, two mice (C11-1 and C11-2), which had been inoculated with tissues from G1 pigs that survived until the end of the experiment (day 42) but also tested positive for *T. gondii* by detection of parasite DNA in their brains. Accordingly, they were the only two mice which gave a seropositivity of greater than 3% with the *T. gondii* IgG ELISA. It is likely that mice, which were euthanised within the first 14 days of the bioassay, which also tested positive for the presence of *T. gondii* DNA in their brains, may only just have started to mount a humoral immune response against the parasite, however, the levels of IgG present were possibly too low to be detected by the ELISA.

Although the *T. gondii* specific ITS1 PCR was carried out using DNA from individual porcine tissues (brain, chop, loin, left tricep, left semitendinosus, diaphragm, heart, tongue and masseter), parasite DNA could not be detected from any tissues. When comparing this result with a similar study carried out in lambs [11], where *T. gondii* DNA was detected from individual tissues by ITS1 PCR, it is likely that the challenge dose of 1000 oocysts may be too low to be detected by this method. Although 1000 M4 oocysts is more likely to reflect that of a natural infection, it appears that a higher challenge dose (500 000 M4 oocysts as used by [11]), may result in a greater chance of detecting parasite DNA directly from the hosts tissue, without the need for mouse bioassay or detection of parasite DNA from bioassay inocula. In addition, direct detection of parasite DNA from porcine tissues used only 1 g of starting material, compared with 50 g of tissue which was prepared for mouse bioassay, therefore, due to the inhomogeneous distribution of parasite tissue cysts there was less chance of detecting the parasite from a smaller 1 g sample. In the current study, detection of parasite DNA from bioassay inocula was tested by two different molecular techniques; the 529 bp *T. gondii* qPCR and the *T. gondii* ITS1 PCR, to determine which test was more sensitive and whether either technique was as sensitive as mouse bioassay (the “gold standard” assay for detection of viable tissue cysts). Although detection of parasite DNA from mouse brains, following bioassay by ITS1 PCR, detected the greatest number of positive samples there was no evidence of a difference between two molecular tests. However, the sensitivity of the ITS1 PCR was slightly higher than the 529 bp qPCR (0.78 and 0.67 respectively). It should also be noted that although these two molecular techniques successfully detect parasite DNA, it is still currently only the mouse bioassay which has the ability to detect viable tissue cysts, and their potential to infect another host. However, perhaps future methodology could employ both molecular detection of the parasite from bioassay inocula in conjunction with mouse bioassay, with a view to reducing the number of mice used within the bioassay, such as magnetic capture qPCR (MC-qPCR) of *T. gondii* DNA as described by [10]. Histological examination of porcine samples was of limited use to detect the parasite in the porcine tissue or to show differences between the groups of infected mice. However, it showed how vaccination protected against the occurrence of lesions in the brain (perivascular foci) after infection. Immmunohistochemical labelling was also ineffective when trying to localize the parasite, as only in two animals tachyzoites-like structures were detected. These results from pathological studies suggest that these techniques are not adequate when studying parasite distribution in studies where no (porcine experiment) or very similar (murine bioassay) lesions are originated.

Overall, in terms of vaccination to reduce viable tissue cysts in meat, these results are promising, as this is the first description of 100% protection against tissue cyst formation in mice following mouse bioassay of tissues from pigs. The results also provide an answer to the question raised by the Food Standards Agency, in a recent report published by the Advisory Committee on the Microbiological Safety of Food (ACMSF), the document states that one of the knowledge gaps relevant to a risk assessment in the UK is: “*vaccines based on live attenuated strains of tachyzoites are effective in reducing morbidity in sheep but it is not known whether vaccination has any effect on the formation of tissue cysts*” [26]. From the current research it is now clear that S48 does have a significant effect in reducing the formation of tissue cysts in pigs. Vaccination of other “high risk” livestock species should be addressed and it has recently been demonstrated that vaccination of sheep with S48 also results in a reduction in the number of ovine tissue samples in which parasite DNA can be detected [11].
